# ACBD5 and VAPB mediate membrane associations between peroxisomes and the ER

**DOI:** 10.1083/jcb.201607055

**Published:** 2017-02

**Authors:** Joseph L. Costello, Inês G. Castro, Christian Hacker, Tina A. Schrader, Jeremy Metz, Dagmar Zeuschner, Afsoon S. Azadi, Luis F. Godinho, Victor Costina, Peter Findeisen, Andreas Manner, Markus Islinger, Michael Schrader

**Affiliations:** 1 Biosciences, University of Exeter, Exeter EX4 4QD, England, UK; 2 Max Planck Institute for Molecular Biomedicine, 48149 Muenster, Germany; 3 Institute for Clinical Chemistry, Medical Faculty Mannheim, University of Heidelberg, 68167 Mannheim, Germany; 4 Institute of Neuroanatomy, Center for Biomedicine and Medical Technology Mannheim, Medical Faculty Mannheim, University of Heidelberg, 68167 Mannheim, Germany

## Abstract

Peroxisomes (POs) and the endoplasmic reticulum (ER) cooperate in cellular lipid metabolism and form tight structural associations, which were first observed in ultrastructural studies decades ago. PO–ER associations have been suggested to impact on a diverse number of physiological processes, including lipid metabolism, phospholipid exchange, metabolite transport, signaling, and PO biogenesis. Despite their fundamental importance to cell metabolism, the mechanisms by which regions of the ER become tethered to POs are unknown, in particular in mammalian cells. Here, we identify the PO membrane protein acyl-coenzyme A–binding domain protein 5 (ACBD5) as a binding partner for the resident ER protein vesicle-associated membrane protein-associated protein B (VAPB). We show that ACBD5–VAPB interaction regulates PO–ER associations. Moreover, we demonstrate that loss of PO–ER association perturbs PO membrane expansion and increases PO movement. Our findings reveal the first molecular mechanism for establishing PO–ER associations in mammalian cells and report a new function for ACBD5 in PO–ER tethering.

## Abstract (precis)

Costello et al. identify ACBD5 and VAPB as key components of a peroxisome–ER tether in mammalian cells. Disruption of this tethering complex leads to reduced peroxisomal membrane expansion and increased peroxisomal movement.

## Introduction

Peroxisomes (POs) are multifunctional organelles that play pivotal roles in the metabolism of lipids and reactive oxygen species and are essential for human health and development (Wanders and Waterham, 2006; Nordgren and Fransen, 2014). These functions require a dynamic spatial organization that permits exchange of metabolites and signals with other organelles such as the ER, mitochondria, lipid droplets, and lysosomes (Chu et al., 2015; Gao and Goodman, 2015; Schrader et al., 2015b). POs collaborate extensively with the ER in the biosynthesis of ether-phospholipids (e.g., myelin sheath lipids) and polyunsaturated fatty acids, and defects in these pathways are linked to neurodegenerative disorders (Wanders and Poll-The, 2015). Furthermore, the ER is involved in PO biogenesis, likely playing a role in the delivery of phospholipids to PO (Raychaudhuri and Prinz, 2008; Hettema et al., 2014). In ultrastructural studies, POs are often found apposed to ER tubules (Novikoff and Shin, 1964) with short electron-dense cross-bridges between isolated POs and associated ER, suggesting an intimate, physical interaction (Zaar et al., 1987). Despite the decades that have passed since PO–ER associations were first observed, we still know little about their formation, structure, and function. In yeast, the EPCON (ER–PO contact) complex and an PO–ER junction complex involving Pex3 and Inp1p required for PO inheritance have been reported, but analogous systems in higher eukaryotes have not been identified (David et al., 2013; Knoblach et al., 2013).

Here, we identify the PO membrane protein acyl-coenzyme A–binding domain protein 5 (ACBD5) as a binding partner for the ER protein VAPB (vesicle-associated membrane protein-associated protein B). We show that ACBD5–VAPB interaction regulates PO–ER associations, the loss of which perturbs PO membrane expansion and increases PO motility. Our findings reveal the first molecular mechanism for establishing PO–ER associations in mammalian cells and a new function for ACBD5 in PO–ER tethering.

## Results and discussion

### Peroxisomal ACBD5 is a binding partner for ER-resident VAPB

**Figure 1.**
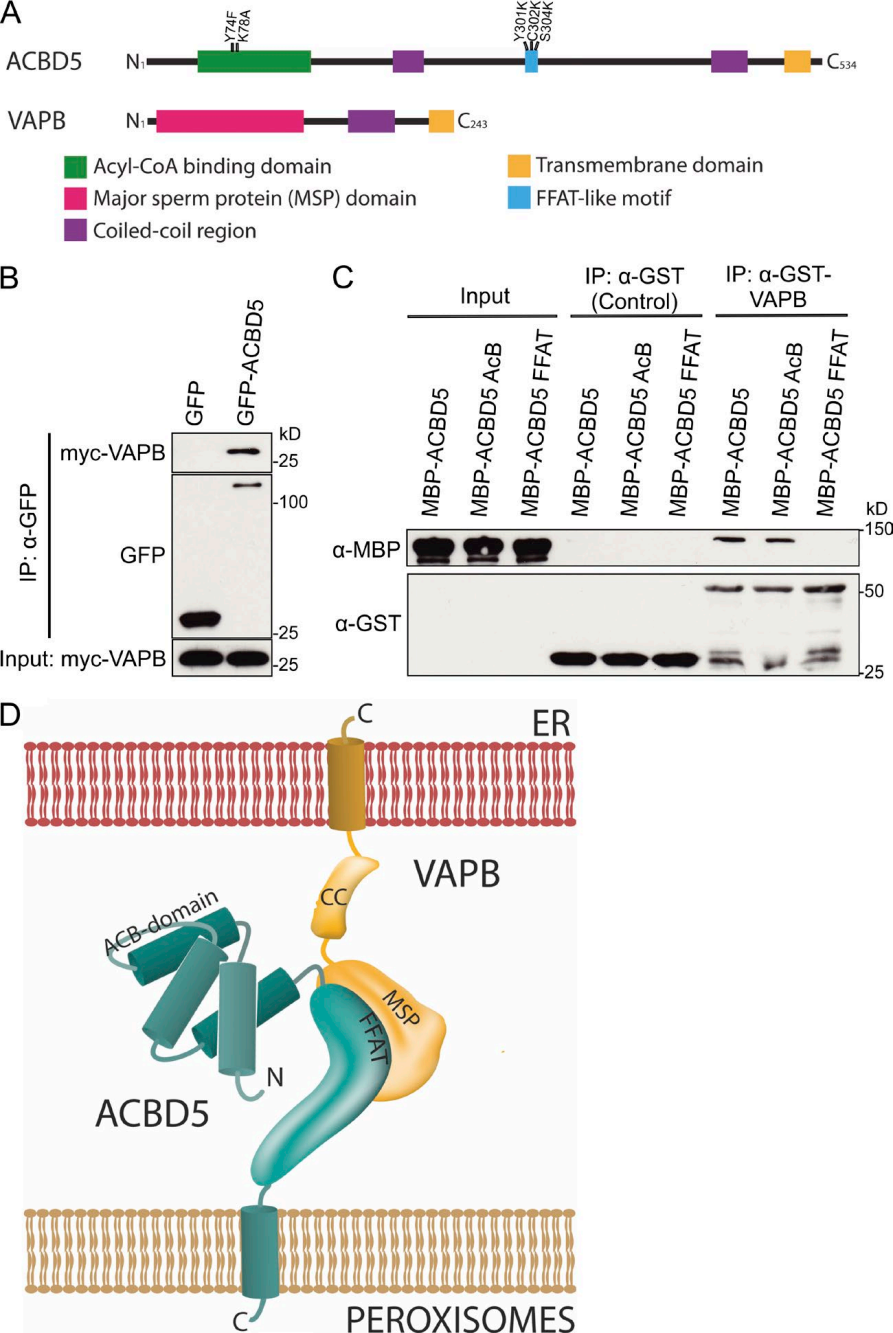
**ACBD5 interacts with VAPB.** (A) Schematic overview of VAPB and ACBD5 domain structure. Mutations in acyl-CoA binding and FFAT-like motifs are indicated. (B) Immunoprecipitation (IP) of GFP-ACBD5 and Myc-VAPB after coexpression in COS-7 cells. GFP was used as control. Samples were immunoprecipitated (GFP-Trap) and immunoblotted using Myc-GFP antibodies. (C) In vitro binding assay using GST-VAPB and MBP fusions of ACBD5 (AcB, mutations in the acyl-CoA binding motif; FFAT, mutations in FFAT motif) expressed in *E. coli*. GST served as control. Samples were immunoprecipitated using GST-Trap and immunoblotted using MBP-GST antibodies. (D) Model of ACBD5–VAPB interaction.

Previous studies identified ACBD5 in highly purified PO fractions and revealed its exclusive PO localization (Islinger et al., 2007; Wiese et al., 2007; Nazarko et al., 2014). To identify potential binding partners of ACBD5, we expressed GFP-ACBD5 in HepG2 cells and performed pull-down studies and mass spectrometry (MS) analysis. Results from three independent experiments identified the ER membrane protein VAPB as a candidate binding partner (Table 1 and Fig. 1 A). In two out of three experiments, we also found enrichment of the closely related protein VAPA (Table 1). ACBD5–VAPB binding was confirmed by immunoprecipitation (IP) after coexpression of GFP-ACBD5 and Myc-VAPB in COS-7 cells (Fig. 1 B). A direct interaction between ACBD5 and VAPB was shown by expressing recombinant versions in *Escherichia coli* and performing in vitro binding assays (Fig. 1 C). Additionally, in a genome-wide protein interaction screen, ACBD5 was among proteins identified as potential VAPB interaction partners (Huttlin et al., 2015).

**Table 1.** Identification of VAPB and VAPA by MS after coimmunoprecipitation with GFP-ACBD5 from HepG2 cells

Accession number	Gene name	Mass	Coverage, maximum	Matched peptides, maximum	Unique peptides, maximum	ACBD5/control ratio, average	Number of experiments detected
		*D*	*%*				
Q528D3	ACBD5	60,092	16.0	8	8	>10	3/3
O95292	VAPB	27,228	51.4	16	14	4.8	3/3
A8KA83	VAPA	27,318	47.9	20	18	7.7	2/3

Results from three experiments. GFP was used as a control. Protein abundance was quantified by label-free peptide counting.

VAPB is a tail-anchored ER membrane protein, which functions as an adaptor in interorganellar lipid exchange and membrane tethering (Lev et al., 2008). VAPB interactions are mediated by its MSP domain (Fig. 1 A), which recognizes FFAT-like motifs (two phenylalanines [FF] in an acidic tract), one of which has recently been predicted in ACBD5 (Murphy and Levine, 2016). In line with this, VAPB–ACBD5 interaction was lost when critical residues in the FFAT-like motif were mutated (Fig. 1 C). In contrast, VAPB–ACBD5 interaction was still observed after mutating residues in the ACBD5 acyl-coenzyme A–binding domain (AcB), shown to abolish acyl-coenzyme A (acyl-CoA) binding (Kragelund et al., 1999; Nazarko et al., 2014). We conclude that VAPB and ACBD5 interact directly via the ACBD5 FFAT-like motif (Fig. 1 D).

### ACBD5 and VAPB mediate PO–ER associations

**Figure 2.**
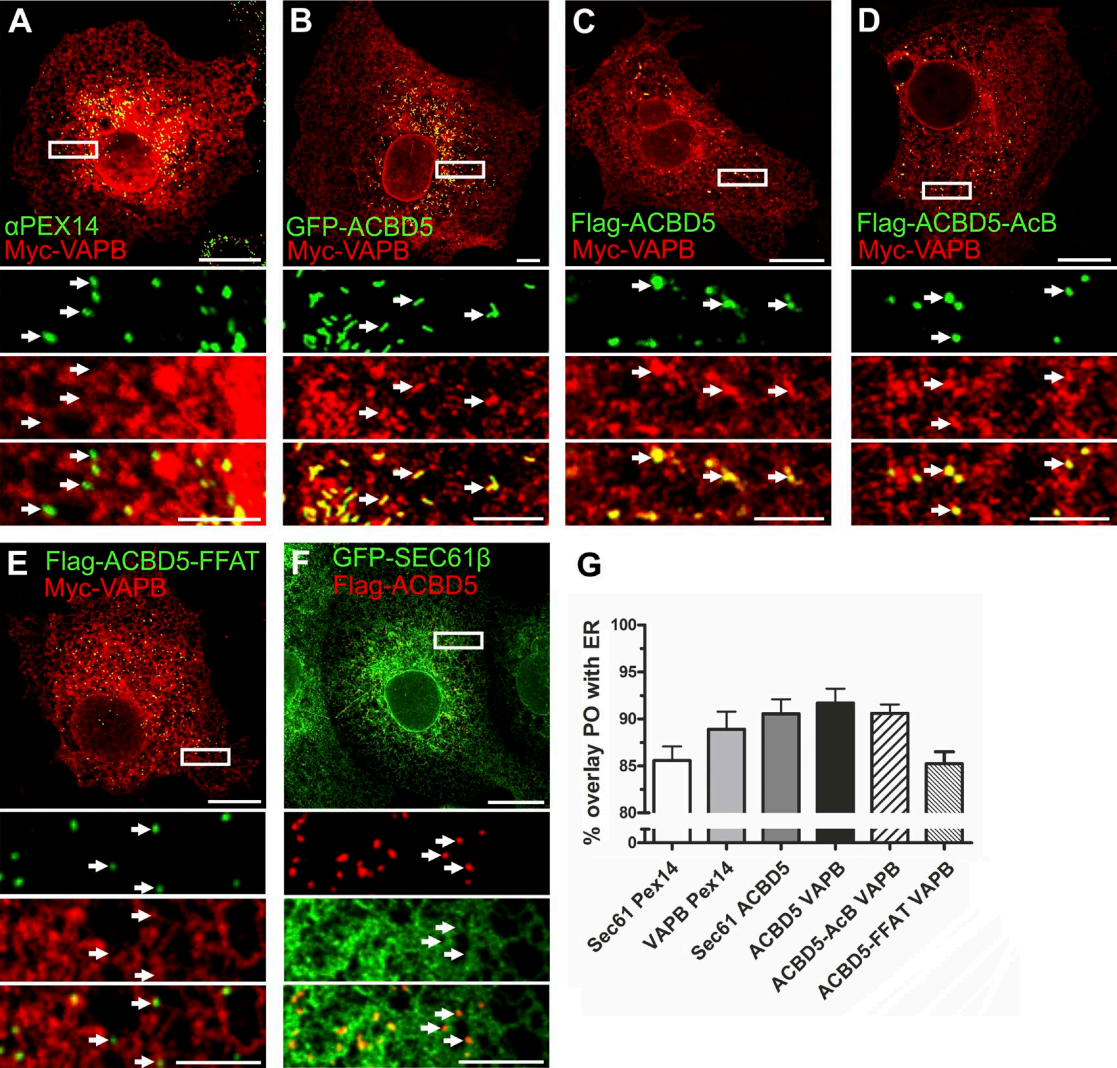
**ACBD5**–**VAPB coexpression increases PO–ER association**. (A–F) COS-7 cells were transfected with Myc-VAPB alone (A) or Myc-VAPB coexpressed with GFP-ACBD5, FLAG-ACBD5, FLAG-ACBD5-AcB, FLAG-ACBD5-FFAT, and GFP-Sec61β (B–F). Myc-VAPB is labeled in red and Pex14/ACBD5 in green, except in F, where ACBD5 is in red (B–D, arrows highlight PO–ER association; A, E, and F, arrows highlight lack of PO–ER association). (G) Quantification of overlap of PO–ER fluorescent signals. Note this analysis has limitations because of the proximity of POs and the ER. αPEX14, PO marker. Data are presented as mean ± SEM. Bars: (main) 20 µm; (insets) 5 µm.

As VAPB is involved in ER–organelle associations (Lev et al., 2008; Peretti et al., 2008; Stoica et al., 2014), we hypothesized that ACBD5 and VAPB act as tethers linking the ER with POs. To test this, we expressed Myc-VAPB and/or GFP-ACBD5/FLAG-ACBD5 in COS-7 cells and monitored PO–ER colocalization by confocal microscopy (Fig. 2). POs are usually in close proximity to the ER (Novikoff and Shin, 1964) but never fully overlap with ER markers (Fig. 2, Sec61β). When both VAPB and ACBD5 were coexpressed, increased association of PO with Myc-VAPB-labeled ER was observed (Fig. 2, B and C). Remarkably, coexpression allowed visualization of discrete PO structures using the ER marker VAPB (compare arrows in Fig. 2, A and B), suggesting these POs are in close contact with the ER. This characteristic PO–ER association was still observed when VAPB was coexpressed with the ACBD5-AcB mutant (Fig. 2 D), but not after coexpression with the ACBD5 FFAT mutant (Fig. 2 E). Although coexpression of ACBD5 with the ER protein Sec61β slightly increased PO–ER overlay (Fig. 2 G), characteristic PO–ER associations were not evident (Fig. 2 F). Quantification of fluorescence signals indicated a slight increase in PO–ER overlap when ACBD5 or VAPB was expressed individually, but overlap was highest after coexpression (Fig. 2 G). In line with this, a mutant ACBD5 localizing to mitochondria increased association of mitochondria with VAPB-labeled ER (Fig. S1). These findings support a role for ACBD5 and VAPB in PO–ER tethering, which we further investigated using ultrastructural and biochemical approaches.

**Figure 3.**
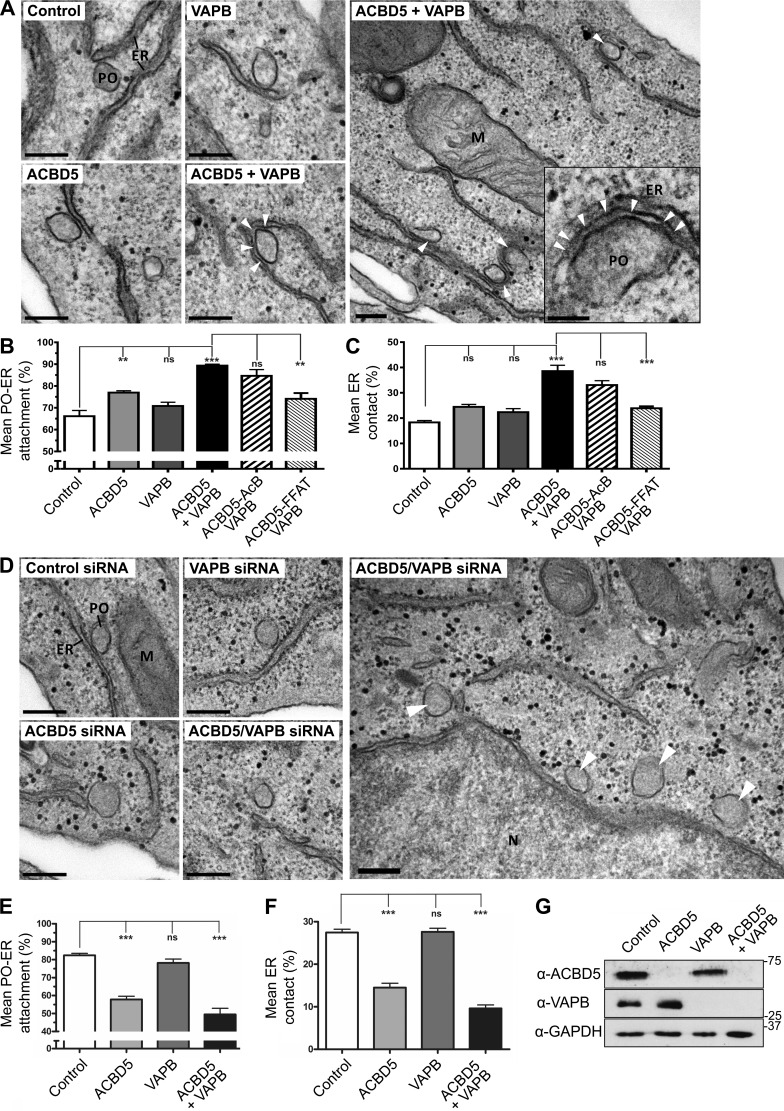
**ACBD5 and VAPB mediate PO–ER association.** (A) Representative electron micrographs of PO–ER associations in COS-7 cells transfected with control vector, ACBD5, VAPB, and ACBD5 + VAPB (arrowheads highlight close ER–PO association). Electron-dense material between the PO–ER membranes is visible (see arrows in enlargement). (B) Quantitative analysis of the mean fraction of POs associated with the ER. (C) Assessment of the mean PO membrane surface in direct contact with the ER membrane. (D) Representative electron micrographs of PO–ER associations in HepG2 cells treated with control, ACBD5, VAPB, and ACBD5 + VAPB siRNAs. Arrowheads in overview mark POs with limited or no ER contact. (E) Quantitative analysis of mean fraction of POs associated with the ER after siRNA treatments. (F) Assessment of mean PO–ER membrane contact after siRNA treatment. (G) Immunoblot showing ACBD5 and VAPB signals after silencing with correspondent siRNAs. GAPDH, loading control. Data were analyzed by one-way analysis of variance with Tukey’s multiple comparison test; ns, not significant; **, P ≤ 0.01; ***, P ≤ 0.001. Error bars represent SEM, with three to six experiments per condition. Bars: (main) 200 nm; (zoom) 50 nm. M, mitochondrion; N, nucleus.

To quantify PO–ER associations at the ultrastructural level, we performed transmission EM with COS-7 cells expressing FLAG-ACBD5, Myc-VAPB, or FLAG-ACBD5/Myc-VAPB (Fig. 3 A). We quantified PO–ER associations using unbiased spatial stereology to (a) determine the mean population of POs in close contact (<15 nm) with the ER (Fig. 3 B, mean attachment) and (b) estimate the proportion of the PO surface closely apposed (<15 nm) to the ER (Fig. 3 C, mean ER contact). A similar approach using manual tracing software was used by others (Cosson et al., 2012; Stoica et al., 2014). When ACBD5 and VAPB were coexpressed, the mean population of PO associated with the ER increased significantly (89.35 ± 0.69% vs. 66.17 ± 2.63% in controls; Fig. 3 B), and the mean ER contact doubled (Fig. 3 C). Both the mean PO–ER attachment and mean ER contact were also slightly increased in cells expressing VAPB or ACBD5 alone (Fig. 3, A–C). Coexpression of VAPB with the ACBD5-AcB mutant was comparable to wild-type, whereas coexpression with the ACBD5-FFAT mutant resulted in a significantly lower increase in both mean ER contact and PO–ER attachment (Fig. 3, B and C; and Fig. S2).

PO–ER associations were confirmed by immunogold-EM of COS-7 cells expressing a GFP-PO targeting signal 1 fusion (GFP-PTS1), which targets the PO matrix. Cells were mock-treated or cotransfected with ACBD5/VAPB and PO identified by anti-GFP antibodies (Fig. S2). In control cells, globular POs were mainly located in close vicinity to the ER (Fig. S2). In cotransfected cells ER membranes were tightly associated with GFP-labeled PO, often covering large portions of the PO surface. Electron-dense structures were visible between the ER and PO membranes, appearing to tether ER with PO (Fig. S2 and Fig. 3 A). The space between ER and PO in cotransfected cells was <15 nm, similar to that observed in other organelle membrane associations (Prinz, 2014).

Similar results regarding PO–ER association were obtained with HepG2 cells (Fig. S2). Here, we silenced ACBD5 and/or VAPB and quantified PO–ER associations by transmission EM (Fig. 3, D–F). Using siRNAs, ACBD5 and VAPB expression was efficiently reduced, without one influencing expression of the other (Fig. 3 G). In controls, 82.40 ± 1.09% of POs were in close contact with the ER, and 27.35 ± 0.77% of the PO surface was closely associated with the ER. Knockdown of either ACBD5 or both ACBD5 and VAPB significantly reduced the fraction of POs associated with the ER (ACBD5: 57.85 ± 1.76%; ACBD5 + VAPB: 49.45 ± 3.45%) and the mean ER contact (ACBD5: 14.52 ± 1.0%; ACBD5 + VAPB: 9.62 ± 0.82%; Fig. 3, D–F). Knockdown of VAPB alone had only a minor effect (Fig. 3, E and F). This may be explained by the ability of ACBD5 to interact with other proteins such as the functionally similar VAPA (Table 1; Murphy and Levine, 2016).

**Figure 4.**
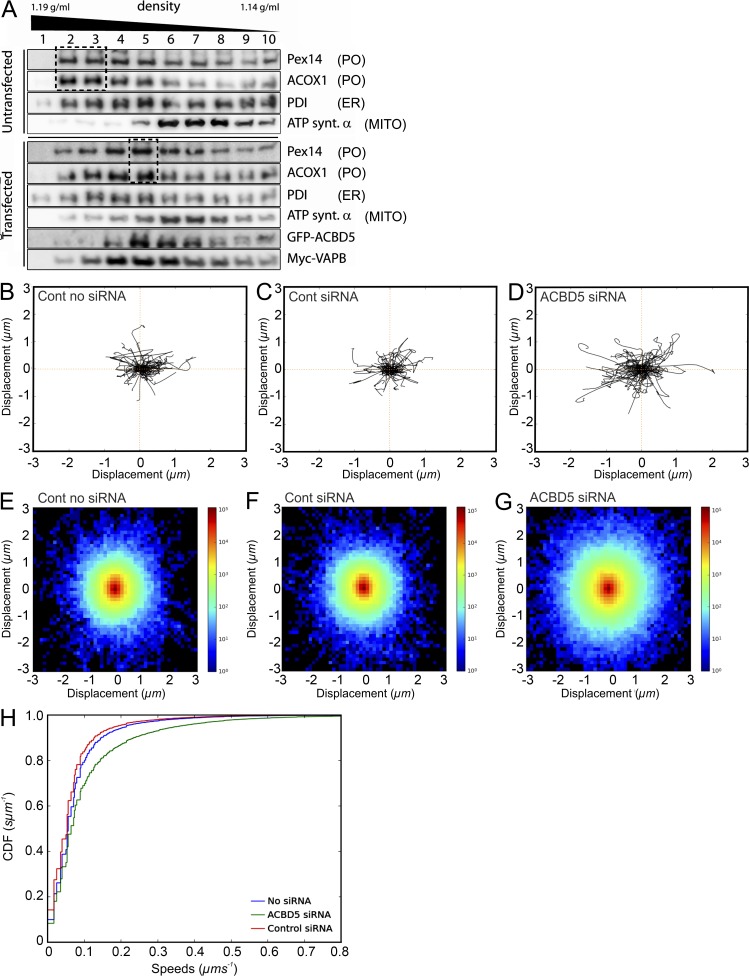
**ACBD5/VAPB interaction influences PO migration in gradients and PO motility in human fibroblasts.** (A) ACBD5/VAPB coexpression alters PO distribution in density gradients. PO-enriched fractions, prepared from HepG2 cells (Control) and cells cotransfected with GFP-rACBD5 and Myc-VAPB, were separated in continuous Nycodenz-gradients. Distribution of organelle markers was assessed by immunoblotting of fractions. Coexpression of ACBD5 and VAPB shifts POs to lower densities, similar to ER markers (compare boxed regions). Pex14 (PO); ACOX1, acyl-CoA oxidase 1 (PO); ATP synt. a, ATP synthase α subunit (MITO); PDI, protein disulfide isomerase (ER). (B–H) Loss of ACBD5 increases PO movement. Human fibroblasts were treated with control (Cont) or ACBD5 siRNA, transfected with GFP-PTS1, and analyzed by live cell imaging (Videos 1 and 2). (B–D) Trajectory plots. 100 PO trajectories were retrieved for each condition and the first 20 time frames plotted starting at a center. (E–G) Density plots. The x and y coordinates of all trajectories ≥20 time frames were pooled and binned in the interval −3,3 µm in x and y directions, using 50 bins. The log-scaled 2D histogram of these points was plotted using “jet” color map. (H) ECDF plots. Instantaneous trajectory speed profiles were estimated by calculating distance moved between each time point in the trajectory. These speeds were pooled and converted to an empirical cumulative distribution function (ECDF). By pooling speeds for all datasets for a given condition, a single ECDF was generated for each (minimum of 38,175 trajectories from 24 videos per condition).

To confirm the morphological results by biochemical analyses, we separated POs from HepG2 cells using a combination of differential and Nycodenz gradient centrifugation. Gradient fractions were collected from untransfected cells and cells cotransfected with Myc-VAPB and GFP-ACBD5 (Fig. 4 A). POs in control gradients were well separated from mitochondria and migrated to higher densities than microsomes (PO fractions 2 and 3 vs. 4 and 5 for ER; Fig. 4 A). When ACBD5 and VAPB were coexpressed, POs and microsomes partially comigrated, showing comparable maxima for marker proteins (fraction 5; Fig. 4 A). These findings suggest a tighter association between both organelles in cells coexpressing ACBD5 and VAPB, creating PO–ER structures with altered buoyant densities, supporting a role for ACBD5 and VAPB in PO–ER association.

### Silencing of ACBD5 increases PO movements

Next, we investigated if an ACBD5-dependent PO–ER association would affect PO motility. Fibroblasts were cotransfected with GFP-PTS1 and ACBD5 siRNA (Fig. 4, B–H; and Videos 1 and 2), and PO motions recorded in individual cells. No differences in PO movements between untreated cells and those treated with control siRNA were observed. In contrast, silencing of ACBD5 resulted in a prominent increase of PO movements (Video 2). To measure movement, POs were automatically detected and tracked using a customized in-house algorithm. The trajectories of 100 randomly sampled POs were plotted to visualize displacements from a central point (Fig. 4, B–D). Displacements increased when ACBD5 was silenced. To extend this analysis, the displacements of all POs from each experimental group were plotted as a 2D histogram (Fig. 4, E–G). In agreement with the first analysis, PO displacement was clearly increased after ACBD5 knockdown. Fig. 4 H displays the empirical cumulative distribution function (ECDF) of the instantaneous PO speeds for each experimental condition. This shows the distribution of the total population of POs, with each point of the curve corresponding to a single movement. A significant increase in the number of moving POs can be observed in ACBD5-silenced cells (mean speed, 100 ± 6 nm/s vs. 70 ± 3 nm/s in controls; ***, P ≤ 0.001; Student’s *t* test). These findings further support a role for ACBD5 in attaching POs to the ER, which appears to restrict PO movement.

### A role for ACBD5–VAPB in PO membrane dynamics

**Figure 5.**
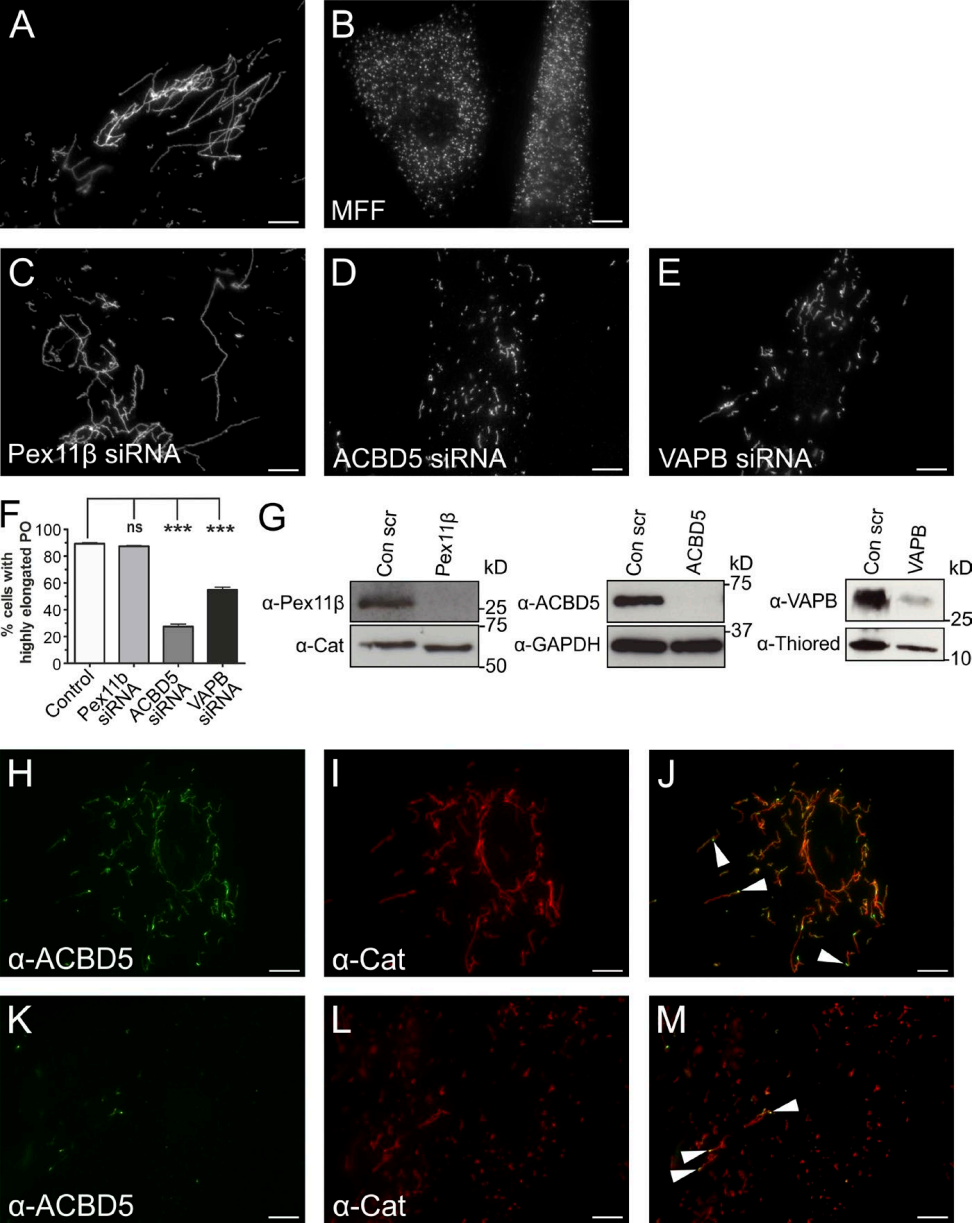
**Loss of ACBD5 or VAPB reduces PO membrane expansion in Mff-deficient fibroblasts.** PO morphology in Mff-deficient fibroblasts (control; A) after reintroduction of Mff (B) or silencing of Pex11β (C), ACBD5 (D), or VAPB (E). Fixed cells were labeled with anti-Pex14 antibodies. (F) Quantification of PO morphology in controls and silenced cells (*n* = 2,500, from three independent experiments). Data are presented as mean ± SEM. ***, P < 0.001; ns, not significant. (G) Immunoblots of cell lysates. Loading controls used were catalase (Cat), GAPDH, and thioredoxin (Thiored). (H–M) Localization of endogenous ACBD5 in Mff-deficient fibroblasts. Fixed cells labeled with anti-ACBD5 and anti-catalase antibodies. Arrowheads denote ACBD5 concentrated at globular POs that give rise to tubular membranes. Bars, 10 µm.

PO can form by growth and division of preexisting organelles (Schrader et al., 2015a). A key protein in this process is Pex11β, which deforms and elongates the PO membrane and activates the GTPase DRP1 for membrane scission (Schrader et al., 1998; Koch et al., 2003; Delille et al., 2010; Williams et al., 2015). DRP1 is recruited to POs by the membrane adaptors Mff and Fis1 (Koch et al., 2005; Gandre-Babbe and van der Bliek, 2008; Otera et al., 2010). Loss of Mff results in highly elongated POs (and mitochondria), which are unable to divide (Shamseldin et al., 2012; Koch et al., 2016; Fig. 5 A). As elongation and growth of the PO membrane requires lipids, likely provided by the ER in a nonvesicular pathway (Raychaudhuri and Prinz, 2008), the pronounced PO elongation observed after loss of Mff suggests a constant transfer of lipids from the ER to POs. We hypothesized that this lipid transfer is mediated by PO–ER contacts. To investigate a role for ACBD5–VAPB in this process, we silenced ACBD5 or VAPB in patient fibroblasts deficient in Mff (Fig. 5). Reintroduction of Mff into nonsilenced cells resulted in formation of numerous spherical POs, restoring the normal phenotype (Fig. 5 B). Although knockdown of Pex11β had no effect, remarkably, silencing of ACBD5, and to a lesser extent VAPB, reduced membrane expansion, resulting in formation of shorter PO membrane tubules and spherical organelles (Fig. 5, C–F). To confirm the alterations were caused by membrane expansion, we quantified the size of PO membranes using EM and observed a significant reduction in mean PO membrane circumference when comparing ACBD5-silenced cells with controls (Fig. S3). This effect was PO specific, as ACBD5 silencing had no impact on the elongated morphology of mitochondria (Fig. S3). Finally, expression of an artificial PO–ER tether restored membrane expansion after ACBD5 silencing and caused hyperelongation of POs in Mff-deficient fibroblasts (Fig. S3).

As POs in Mff-deficient cells are division incompetent, ACBD5 siRNA-mediated alterations in PO morphology are unlikely to result from PO membrane fission, which would increase PO abundance as shown for reintroduction of Mff. We also localized endogenous ACBD5 in Mff-deficient cells (Fig. 5, H–J; and Fig. S3). ACBD5 was predominantly located at globular PO membrane domains, which gave rise to tubular membrane extensions. This corroborates previous findings (Delille et al., 2010) and is further exemplified in cells silenced for ACBD5, where any residual ACBD5 is found at globular domains of elongated POs (Fig. 5, K–M). In contrast to ACBD5, Pex11β distributes mainly to tubular PO extensions (Delille et al., 2010). We thus assume a direct role for ACBD5 in PO fission is unlikely and suggest that loss of PO–ER association impacts PO membrane elongation, likely because of reduced transfer of membrane lipids. PO membrane biogenesis requires lipid transfer from the ER, likely using a nonvesicular pathway (Raychaudhuri and Prinz, 2008). Organelle tethering can facilitate lipid transfer (Prinz, 2010), although the exact mechanisms and what drives lipid transfer are unknown. Based on data from other membrane contact studies, additional lipid-transfer proteins are likely involved (Lev, 2010). In addition to lipid transfer for membrane proliferation, the PO–ER tether could also facilitate metabolic cooperation in the biosynthesis of ether phospholipids and polyunsaturated fatty acids, which require dual PO–ER activity (Shai et al., 2015).

We show here that association of PO and ER is mediated by interaction of the PO membrane protein ACBD5 and the ER protein VAPB. Both proteins are anchored in the respective organelle membrane via a C-terminal transmembrane domain with N termini projecting into the cytoplasm (Fig. 1 D). VAPB is known to mediate ER–organelle contacts (Lev et al., 2008), making VAPB and ACBD5 plausible candidates for mediating PO–ER interactions. Several lines of evidence support this: (a) ACBD5 and VAPB directly interact in IP assays via a FFAT-like motif in ACBD5; (b) ACBD5 and VAPB coexpression increases PO–ER associations in morphological and biochemical studies, and ACBD5 and VAPB knockdown results in reduced PO–ER interactions; (c) loss of ACBD5 increases PO movements; and (d) loss of ACBD5 or VAPB perturbs PO membrane expansion. In conclusion, our findings reveal the first molecular mechanism for establishing PO–ER associations in mammalian cells.

Recently, a role for ACBD5 and its fungal orthologue, ATG37, in phagophore formation during pexophagy was suggested (Nazarko et al., 2014). Here, we reveal a novel function in PO–ER tethering. ACBD5 can also interact with activated fatty acids, pointing toward a role in lipid metabolism. We show the acyl-CoA–binding domain is not essential for PO–ER tethering, although we cannot exclude a role in modulating the interaction. As POs and the ER cooperate in lipid metabolism, it is intriguing to speculate that ACBD5 fulfills multiple functions, coordinating PO–ER lipid transfer with PO lipid metabolism and biogenesis and turnover.

Both ACBD5 and VAPB are linked to neuropathological disorders (Kim et al., 2010; Abu-Safieh et al., 2013). Mutations in VAPB cause a dominantly inherited form of amyotrophic lateral sclerosis. Thus, dysregulation of the PO–ER associations described here may contribute to some pathological features of amyotrophic lateral sclerosis or other neurodegenerative disorders. Therefore, information on the underlying mechanisms of the PO–ER association could be important for understanding pathogenic processes in disease states. The results described here, which show that ACBD5 and VAPB mediate PO–ER associations, will facilitate future studies on the role of PO–ER associations in several processes, including lipid metabolism, phospholipid exchange, PO biogenesis, and autophagy.

## Materials and methods

### Plasmids and antibodies

For initial cloning of human genes, total RNA was extracted from HepG2 cells using TRIzol reagent, reverse transcribed into cDNA, and used as a PCR template. The artificial PO–ER tether (PO-mRFP-ER) was generated based on a previously published MITO-ER tether (Csordás et al., 2006). The MITO-ER tether contains an N-terminal MITO targeting signal from AKAP1 fused to mRFP with a C-terminal ER localization signal for yeast UBC6. In the PO-ER tether, the N-terminal MITO targeting signal was replaced with the N-terminal 44 residues of human Pex3, which has been shown to be sufficient for PO targeting (Fransen et al., 2001). See Table S1 for details of plasmids used in this study, Table S2 for plasmids generated in this study, and Table S3 for details of primers used. Site-directed mutagenesis to generate point mutations was done using the QuikChange Site-Directed Mutagenesis kit (Agilent Technologies). All constructs were confirmed by sequencing (Eurofins Genomics). Details on siRNA used in this study can be found in Table S4. Details on antibodies can be found in Table S5.

### Cell culture and transfection

COS-7 (African green monkey kidney cells, CRL-1651; ATCC), HepG2 (human hepatoblastoma cells, HB-8065; ATCC), Mff-deficient (provided by F.S. Alkuraya, King Faisal Specialist Hospital and Research Center, Riyadh, Saudi Arabia), and human control (C109) fibroblasts (provided by H. Waterham, Academic Medical Center, University of Amsterdam, Amsterdam, Netherlands) were cultured in DMEM, high glucose (4.5 g/l) supplemented with 10% FBS, 100 U/ml penicillin, and 100 µg/ml streptomycin at 37°C with 5% CO_2_ and 95% humidity. COS-7 and HepG2 cells were transfected using diethylaminoethyl-dextran (Sigma-Aldrich) or Lipofectamine (Invitrogen, Thermo Fisher Scientific). Fibroblasts were transfected by microporation using the Neon Transfection System (Thermo Fisher Scientific) following the manufacturer’s protocol. In short, cells (seeded 24 h before transfection) were washed once with PBS and trypsinized using TrypLE Express. Trypsinized cells were resuspended in complete medium, pelleted by centrifugation, and washed with PBS. The cells were once again centrifuged and carefully resuspended in 110 µl buffer R. For each condition, 4 × 10^5^ cells were mixed with the DNA construct (5–10 µg) or with 50–100 nM siRNA (Table S4). Cells were microporated using a 100 µl neon tip with the following settings: 1,400 V, 20 ms, one pulse. Microporated cells were immediately seeded into plates with prewarmed complete medium without antibiotics and incubated at 37°C with 5% CO_2_ and 95% humidity. Cell lysates to monitor efficiency of silencing were prepared as described under IP.

### Immunofluorescence and microscopy

Cells were processed for immunofluorescence 24 or 48 h after transfection. Cells grown on glass coverslips were fixed with 4% paraformaldehyde in PBS, pH 7.4, permeabilized with 0.2% Triton X-100, and incubated with primary and secondary antibodies as described previously (Bonekamp et al., 2013; Table S5). Cell imaging was performed using an IX81 microscope (Olympus) equipped with an UPlanSApo 100×/1.40 oil objective (Olympus). Digital images were taken with a CoolSNAP HQ2 CCD camera and adjusted for contrast and brightness using the Olympus Soft Imaging Viewer software and MetaMorph 7 (Molecular Devices). Confocal images were obtained using a Leica Biosystems SP8 equipped with an argon laser (488 nm), a DPSS561 laser (561 nm), an HC PL APO 63×/1.3 oil objective, an HC PL APO 100×/1.44 oil objective, and hybrid detectors.

Analyses of PO–ER fluorescence overlap were performed using a custom Python implementation of Pearson and Manders colocalization measures, which used the Numpy and Scikit image libraries (van der Walt et al., 2011, 2014) In brief, after loading, images were split into red and green channels. Cell regions of interest were manually defined, and Otsu thresholding was used to calculate percentage of overlap of foreground pixels and the Pearson and Manders colocalization measures.

For live-cell imaging, fibroblasts were cotransfected with GFP-PTS1 and ACBD5 siRNA 48 h before imaging and plated in 3.5-cm-diameter glass bottom dishes (Cellview; Greiner BioOne). Before image acquisition, a controlled-temperature chamber was set-up on the microscope stage at 37°C, as well as an objective warmer. During image acquisition, cells were kept at 37°C and in CO_2_-independent medium (Hepes buffered). For fibroblasts, 250 stacks of nine planes (0.5-µm thickness and 100-ms exposure) were taken in a continuous stream. All conditions and laser intensities were kept between experiments. Live-cell imaging data were collected using an IX81 microscope equipped with a CSUX1 spinning disk head (Yokogawa), CoolSNAP HQ2 CCD camera, and 60×/1.35 oil objective. Digital images were taken and processed using VisiView software (Visitron Systems).

### PO motility measurements

POs were automatically detected and tracked using a customized in-house algorithm. In brief, each image was filtered using a scale-space Laplace of Gaussian filtering approach (Lindeberg, 1994, 1998) over scales corresponding to the size range of POs. After filtering, a threshold was determined using the median absolute deviation as a robust estimator of the background level (Murtagh and Starck, 2000) and applied to the filtered image to segment POs. Once detected, POs were tracked using a global optimization solution to the linear assignment problem using a modified version of the Jonker–Volgenant algorithm (Jonker and Volgenant, 1987). Tracking results were manually verified for accuracy. For PO trajectory plots, 100 trajectories were retrieved for each condition by randomly selecting approximately four trajectories at least 20 time frames long from each dataset. Next, the trajectories were centered such that each trajectory started at (0,0) and subsequently smoothed applying a simple moving-mean algorithm using a Hann window. The first 20 points along these trajectories were then plotted. For density plots, all trajectories of at least 20 time frames were smoothed as described for the trajectory plots, and subsequently, the x and y coordinates of the centered trajectories were pooled and binned in the interval −3,3 µm in both x and y directions, using 50 bins, corresponding to a bin width of 0.12 µm. The log-scaled 2D histogram of these points using the aforementioned bins was plotted using the “jet” color map. For ECDF plots, instantaneous trajectory speed profiles were estimated by calculating the distance moved between each time-point in the trajectory. These speeds were then pooled and converted into an ECDF. By pooling the speeds for all datasets for a given condition a single ECDF for each condition was generated.

### EM and spatial stereology

For transmission EM, monolayers of COS-7, HepG2 cells and Mff-deficient human skin fibroblasts were fixed in 0.5% glutaraldehyde in 0.2 M Pipes buffer, pH 7.2, for 15 min at room temperature. Cells were then scraped from the culture dish in a small volume of the fixative before pelleting (10 min at 17,000 *g*). After three washes in buffer, the cell pellet was fragmented and postfixed for 1 h in 1% osmium tetroxide (reduced with 1.5% wt/vol potassium ferrocyanide) in 0.1 M sodium cacodylate buffer, pH 7.2. After a series of washes in distilled water (3 × 5 min), the pellet fragments were dehydrated through an ethanol gradient and embedded in Durcupan resin (Sigma-Aldrich). 80-nm ultrathin sections were collected on pioloform-coated 100-mesh copper EM grids (Agar Scientific) and contrasted with lead citrate before imaging using a JEOL JEM 1400 transmission electron microscope operated at 120 kV. For quantification of the extent of PO–ER surface contacts, POs were sampled (*n* = 38–64 POs per experimental condition and grid; mean of 47) by scanning the EM grids systematic uniform random (SUR) and imaging of positively identified PO profiles within the scanning band at a nominal magnification of 80,000 using a digital camera (ES 100W CCD; Gatan). SUR sampling ensured unbiased selection of profiles from the total population of cells regardless of being transfected or nontransfected. Organelle profiles were identified as POs by the presence of a single membrane, a homogenous fine-granular matrix, and profile size. Sampled PO profiles had sizes between 259.92 ± 13.55 nm and 234.99 ± 10.74 nm along the longest axis and 150.28 ± 5.78 nm and 123.72 ± 3.83 nm along the shortest axis in HepG2 and COS-7 cells, respectively (for sizing, *n* = 50 POs per cell line; error = SEM). The morphology and size of sampled POs was similar to POs labeled with GFP-PTS1 by immunogold EM. To estimate the mean fraction of total PO membrane surface in direct contact with the ER, a stereological approach by line intersection counting was used (Weibel, 1979). SUR sampled micrographs were opened in Photoshop CS6, positioned randomly in relation to a square lattice grid (line spacing 184 nm), and intersections with PO membranes counted. Intersections were classified as direct membrane contact (defined as “attachment” in Fig. 3 B) if there was <15 nm distance between PO and ER membranes (yielding between 135 and 335 total intersection counts per experiment). The mean PO membrane circumference in Mff-deficient fibroblasts was also estimated by line intersection counting on SUR sampled PO profiles as described for the measurement of the mean fraction of total membrane surface (*n* = 53–75 POs [mean of 61] and 178–556 total intersection counts per experiment). Statistical analyses were performed in GraphPad Software Prism 5 using a one-way analysis of variance followed by Tukey’s multiple comparison test as well as Lilliefors normality test followed by two-tailed *t* test.

Cryo–immuno-EM was performed according to the Tokuyasu method (Slot and Geuze, 2007). Cells were initially fixed by 2% paraformaldehyde and 0.2% glutaraldehyde in 0.1 M PHEM buffer, pH 7.4. The samples were washed with buffer and embedded in cubes with 10% gelatin. For cryoprotection, the cells were infiltrated overnight in pure 2.3 M sucrose. Blocks were mounted on pins and frozen in N_2_ liquid. Ultrathin cryosections of 50 nm were cut at −110°C in a cryoultramicrotome (UC6; Leica Biosystems). The sections were labeled with anti-GFP antibody and detected by 15 nm protein A–gold conjugate (CMC). Analysis and documentation of the samples were performed at 80 kV at a transmission electron microscope (Tecnai 12 BioTwin; FEI). Selected areas were documented with Ditabis imaging plates.

### IP

For IP experiments GFP-tagged ACBD5 and Myc-tagged VAPB were expressed in COS-7 cells. After 48 h, cells were washed in PBS and then lysed in ice-cold lysis buffer (25 mM Tris-HCl, pH 7.5, 150 mM NaCl, 1% NP-40, 1 mM PMSF, and protease inhibitor cocktail), undissolved material was pelleted by centrifugation at 15,000 *g*, followed by a second centrifugation step at 100,000 *g*. Clarified lysates were then mixed with GFP-TRAP magnetic agarose beads (ChromoTek) and incubated for 2 h at 4°C. Beads were subsequently washed extensively with lysis buffer and bound proteins eluted with Laemmli buffer. Immunoprecipitates and total lysates were analyzed by Western immunoblotting. For in vitro binding assays, GST-VAPBΔTMD and MBP-ACBD5ΔTMD constructs were expressed in BL21 Rosetta (DE3) cells (EMD Millipore) induced with 1 mM IPTG for 4 h. Cells were pelleted by centrifugation at 5,000 *g* for 10 min and cell pellets resuspended in ice-cold *E. coli* lysis buffer (50 mM Tris-HCl, pH 7.5, 300 mM NaCl, 0.1% NP-40, 1 mM PMSF, and protease inhibitor cocktail). Cells were disrupted by sonication, and the 15,000 *g* supernatant was incubated with GST-TRAP agarose beads (ChromoTek) for 2 h at 4°C. Beads were then washed extensively with *E. coli* lysis buffer and bound proteins eluted using Laemmli buffer. Immunoprecipitates and total lysates were subsequently analyzed by Western immunoblotting.

### GFP IP for MS analysis

For GFP IP, 3 × 10^7^ HepG2 cells were transfected with EGFP-rACBD5 or an EGFP-only vector using polyethyleneimine (PEI) as a transfection reagent (see fractionation experiments for more details). After 24 h, transfection efficiency was controlled by fluorescence microscopy. If transfection efficiency surpassed 50%, cells were harvested in lysis buffer (10 mM Tris/Cl, pH 7.5, 150 mM NaCl, 0.5 mM EDTA, and 2% NP-40) and homogenized by shearing through a syringe and needle (27G). After 30-min incubation on ice, unsolubilized material was removed by centrifugation at 100,000 *g*_av_ for 30 min at 4°C. Finally, the NP-40 concentration of the supernatant was adjusted to 1% using dilution buffer (10 mM Tris/Cl, pH 7.5, 150 mM NaCl, and 0.5 mM EDTA). In parallel, 75 µl of GFP-TRAP_A slurry (ChromoTek) was equilibrated according to the protocol supplied by the manufacturer. Subsequently, the GFP-TRAP agarose beads were mixed with the lysates and incubated over night at 4°C on a rotating wheel. Thereafter, the lysates were centrifuged at 2,500 *g*_av_ for 2 min at 4°C and the supernatant discarded. The remaining agarose beads were washed twice with dilution buffer containing 1 M NaCl. After a last washing step with normal lysis buffer, proteins were eluted from the agarose beads by direct incubation in SDS Laemmli buffer.

### Electrophoresis and in-gel digestion for MS

All IP samples (GFP-TRAP) were heated to 95°C for 5 min and cooled on ice before loading onto NuPAGE 4–12% Bis-Tris Gels (Thermo Fisher Scientific). SDS-PAGE was performed according to the manufacturer’s specification. Proteins were fixed within the polyacrylamide matrix by incubating the entire gel in 5% acetic acid in 1:1 (vol/vol) water/methanol for 30 min. After Coomassie staining (60 min), the gel slab was rinsed with water (60 min), and each lane was excised and cut from top to bottom into small pieces. Subsequently, the proteins were in-gel destained (100 mM ammonium bicarbonate/acetonitrile 1:1 vol/vol), reduced (10 mM DTT), alkylated (50 mM iodoacetamide), and finally trypsin-digested by overnight incubation at 37°C. The generated peptides were collected from the gel pieces, which were further subjected to a peptide extraction step with an acidic (1.5% formic acid) acetonitrile (66%) solution. Both peptide-containing samples were combined and dried in a vacuum centrifuge.

### MS

Dried peptides were redissolved in 0.1% trifluoroacetic acid and loaded on a C18 precolumn (Acclaim; Dionex) using a RSLCnano HPLC system (Dionex). Peptides were then eluted with an aqueous-organic gradient, resolved on a C18 column (Acclaim; Dionex) with a flow rate of 300 nl/min, and electrosprayed into a LTQ Orbitrap XL mass spectrometer (Thermo Fisher Scientific). A Triversa Automate (Advion Biosciences) was used as ion source. Each scan cycle consisted of one Fourier transform mass spectrometry full scan and up to seven ion trapping mass spectrometry–dependent MS/MS scans of the seven most intense ions. Dynamic exclusion (30 s), mass width (10 ppm), and monoisotopic precursor selection were enabled. All analyses were performed in positive ion mode. Extracted MS/MS spectra were searched against the Uniprot/Swissprot database using the PEAKS search engine (Bioinformatics Solutions, Inc.) accepting common variable modifications and one missed tryptic cleavage. Peptide tolerance was ±10 ppm, and MS/MS tolerance was ±0.5 D.

### Subcellular fractionation experiments

HepG2 cells were cultivated to 80% confluency in DMEM, low glucose and subsequently transfected with expression plasmids encoding Myc-VAPB and EGFP-rACBD5. Cells were transfected with PEI (25 kD; Polysciences). 1 µg of each plasmid was mixed with a 1 mg/ml PEI solution in a ratio 1:5 or mock transfected with a PEI solution without DNA. The transfection solution was further diluted in serum-free DMEM (1:8.5) and incubated for 15 min at room temperature before transfection. 24 h after transfection, HepG2 cells seeded on coverslips were withdrawn from the cultures and transfection efficiency determined by immunofluorescence analysis. In parallel, the remaining cells were harvested and centrifuged at 500 *g*_av_. A protocol to separate and isolate POs from transfected HepG2 cells was developed by adapting published procedures (Völkl and Fahimi, 1985; Islinger et al., 2007). After a washing step with PBS, the HepG2 cell pellet was suspended in ice-cold homogenization buffer (250 mM sucrose, 5 mM MOPS, 1 mM EDTA, 2 mM PMSF, 1 mM DTT, and 1 mM E-aminocaproic acid, pH 7.4) and cells disrupted by shearing through a syringe and needle (27G). Nuclei and remaining cellular debris were collected by centrifugation at 600 *g*_av_ for 10 min at 4°C. The resulting postnuclear supernatant was subjected to a second centrifugation at 2,000 *g*_av_ for 15 min at 4°C to yield the pellet of the “heavy” mitochondrial fraction. The corresponding supernatant was centrifuged at 20,000 *g_av_* for 20 min at 4°C. The resulting PO-enriched pellet of the “light” mitochondrial fraction was resuspended in homogenization buffered, overlayed onto a linear Nycodenz gradient ranging from 1.14 to 1.19 g/ml density, and centrifuged for 3 h at 100,000 *g*_av_ in a vertical rotor. After centrifugation, 12 fractions of equal volume were collected and organelles enriched by another pelleting step. After determining protein concentration, separations were analyzed by immunoblotting applying 5 µg of protein per lane to the gels.

### Statistical analyses

Unless otherwise indicated, a two-tailed, unpaired *t* test was used to determine statistical differences against the indicated group (*, P < 0.05; **, P < 0.01; ***, P < 0.001). For quantitative analyses of PO morphology (Figs. 5 F and S3), a minimum of 500 cells were examined per condition, and organelle morphology was microscopically assessed in at least three independent experiments. Data are presented as mean ± SEM.

### Online supplemental material

Fig. S1 shows that when ACBD5 is targeted to mitochondria, ER–mitochondria interactions are increased. Fig. S2 shows PO–ER associations using immunogold EM, that ACBD5–VAPB overexpression increases PO–ER interactions in HepG2 cells, and representative electron micrographs for the data shown in Fig. 3 (B and C). Fig. S3 shows that mitochondria are unaffected when ACBD5 is silenced in Mff-deficient cells and that peroxisomal membrane expansion is reduced when measured by EM. Fig. S3 also shows that an artificial PO–ER tether restores membrane expansion when ACBD5 is silenced. Video 1 shows peroxisomal movement in control fibroblasts. Video 2 shows increased peroxisomal movement in fibroblasts silenced for ACBD5. Table S1 shows plasmids used in this study. Table S2 shows plasmids generated in this study. Table S3 shows primers used in this study. Table S4 shows siRNAs used in this study. Table S5 shows primary and secondary antibodies used in this study.

## Supplementary Material

Supplemental Materials (PDF)

Video 1

Video 2
