# Supplementary material for: ACBD5 and VAPB mediate membrane associations between peroxisomes and the ER
Source: J Cell Biol. 2017 Feb;216(2):331–42. doi: 10.1083/jcb.201607055 (PMC5294785; doi:10.1083/jcb.201607055)
Supplement: Supplemental Materials (PDF) [file JCB_201607055_sm.pdf]

Costello et al., https://doi.org/10.1083/jcb.201607055

THE JOURNAL OF CELL BIOLOGY

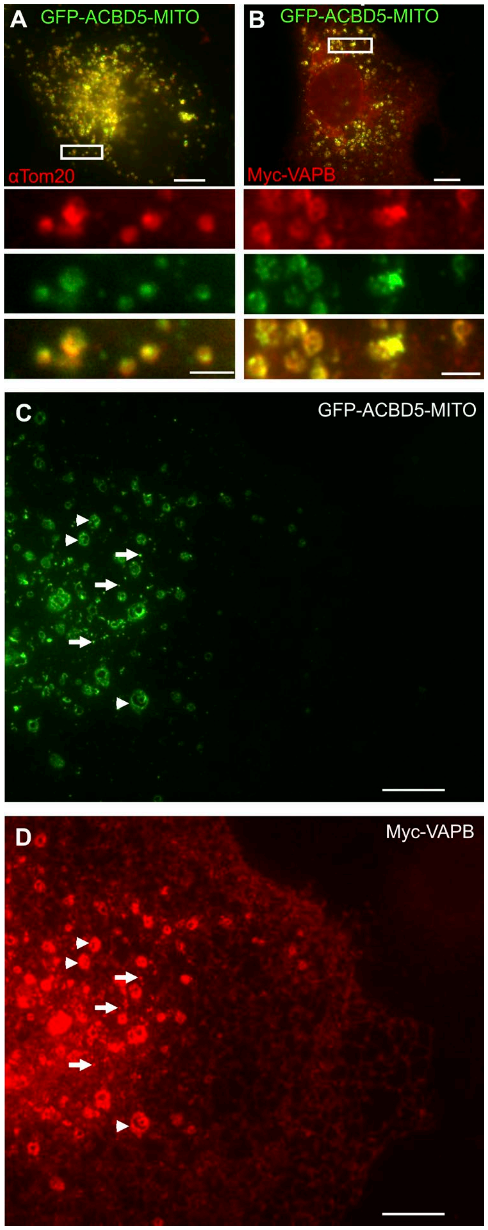

Figure S1. **ACBD5 targeted to mitochondria increases mitochondria–ER associations.** (A) GFP-ACBD5-MITO is targeted to mitochondria and colocalizes with the mitochondrial membrane marker TOM20. Mitochondrial mistargeting of ACBD5 is achieved by mutations in the C-terminal tail region. (B) Coexpression of Myc-VAPB and GFP-ACBD5-MITO results in increased association of mitochondria with ER VAPB. (C and D) Higher-magnification view. Arrows highlight MITO-ER colocalization. Occasionally, GFP-ACBD5-MITO is still targeted to POs, which are also associated with the ER (arrowheads). Note that expression of GFP-ACBD5-MITO alters mitochondrial morphology. Bars: (main) 10 µm; (insets) 2.5 µm.

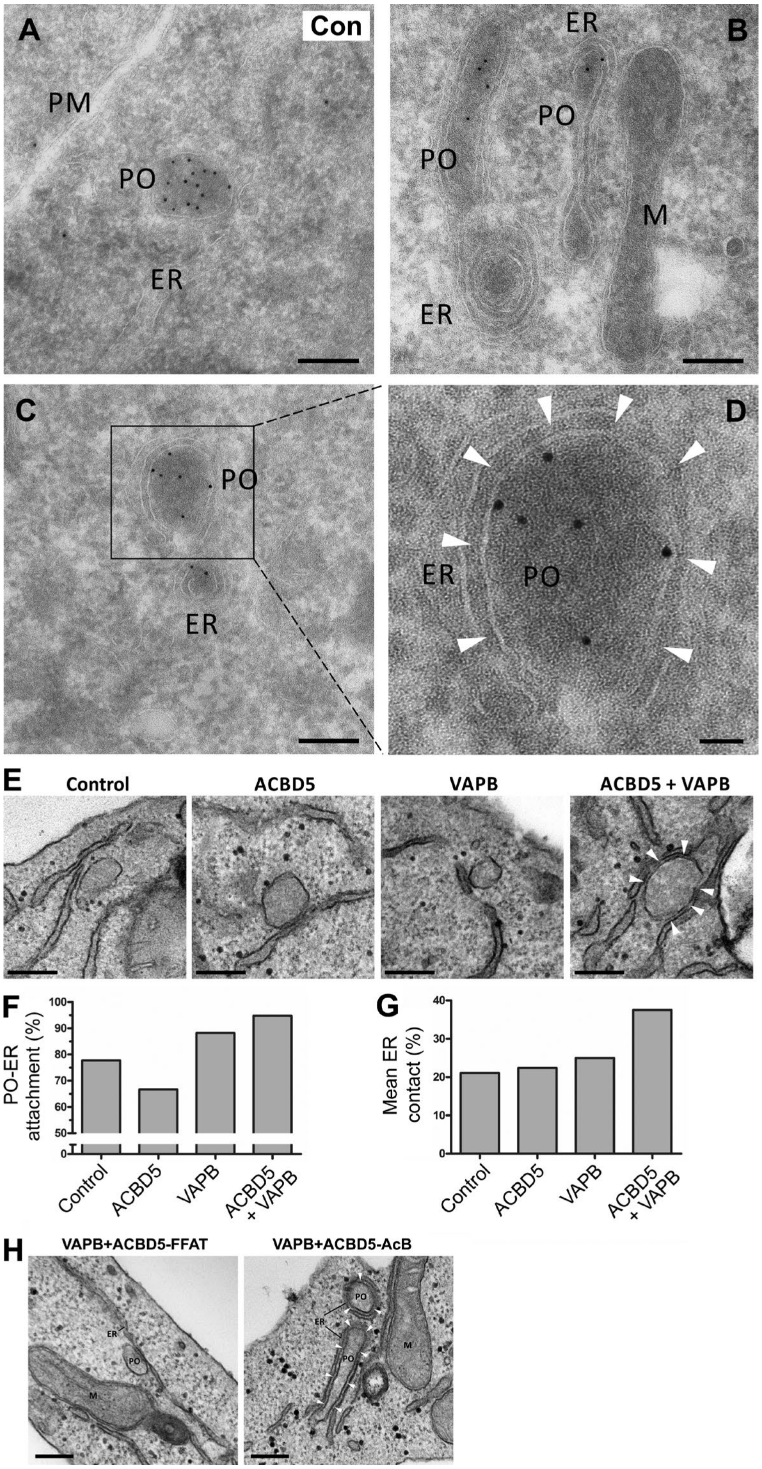

Figure S2. **ACBD5 and VAPB increase PO–ER association.** (A–D) Ultrathin cryosections labeled for GFP protein, detected by 15 nm protein A gold. (A) In mock-treated COS-7 cells (stably expressing the PO marker GFP-PTS1), PO profiles can be identified by GFP labeling, which is mainly located in close vicinity to the ER (control). (B–D) In ACBD5–VAPB cotransfected cells, GFP-labeled POs are closely associated with ER membranes and are partially enclosed (see arrowheads in D). (D) Higher magnification of dashed area in C. Note the presence of electron-dense material between the ER and PO membranes (see Fig. 3). M, mitochondrion. PM, plasma membrane. Bars: (A–C) 200 nm; (D) 50 nm. (E) Representative electron micrographs of PO–ER associations in HepG2 cells transfected with control, ACBD5, VAPB, and ACBD5 + VAPB constructs. Note the increased membrane association in cotransfected cells (arrowheads). Bars, 200 nm. (F) Quantitative analysis of the mean fraction of PO associated with ER in the different samples. (G) Assessment of the mean PO membrane surface in direct contact with ER membrane (see Materials and methods). It should be noted that the number of POs associated with the ER in HepG2 cells under control conditions is higher than in COS-7 cells. This may be because of higher metabolic rates and protein turnover in hepatocytes compared with fibroblasts and the important role of hepatocytes in lipid metabolism, which is supposed to require metabolic cooperation between POs and the ER. (H) Electron micrographs of PO–ER associations in COS-7 cells cotransfected with VAPB and either ACBD5-FFAT or ACBD5-AcB mutants. Note the increased membrane association in ACBD5-AcB cotransfected cells (arrowheads), whereas ACBD5-FFAT–transfected cells show no alterations. Bars, 200 nm. M, mitochondrion.

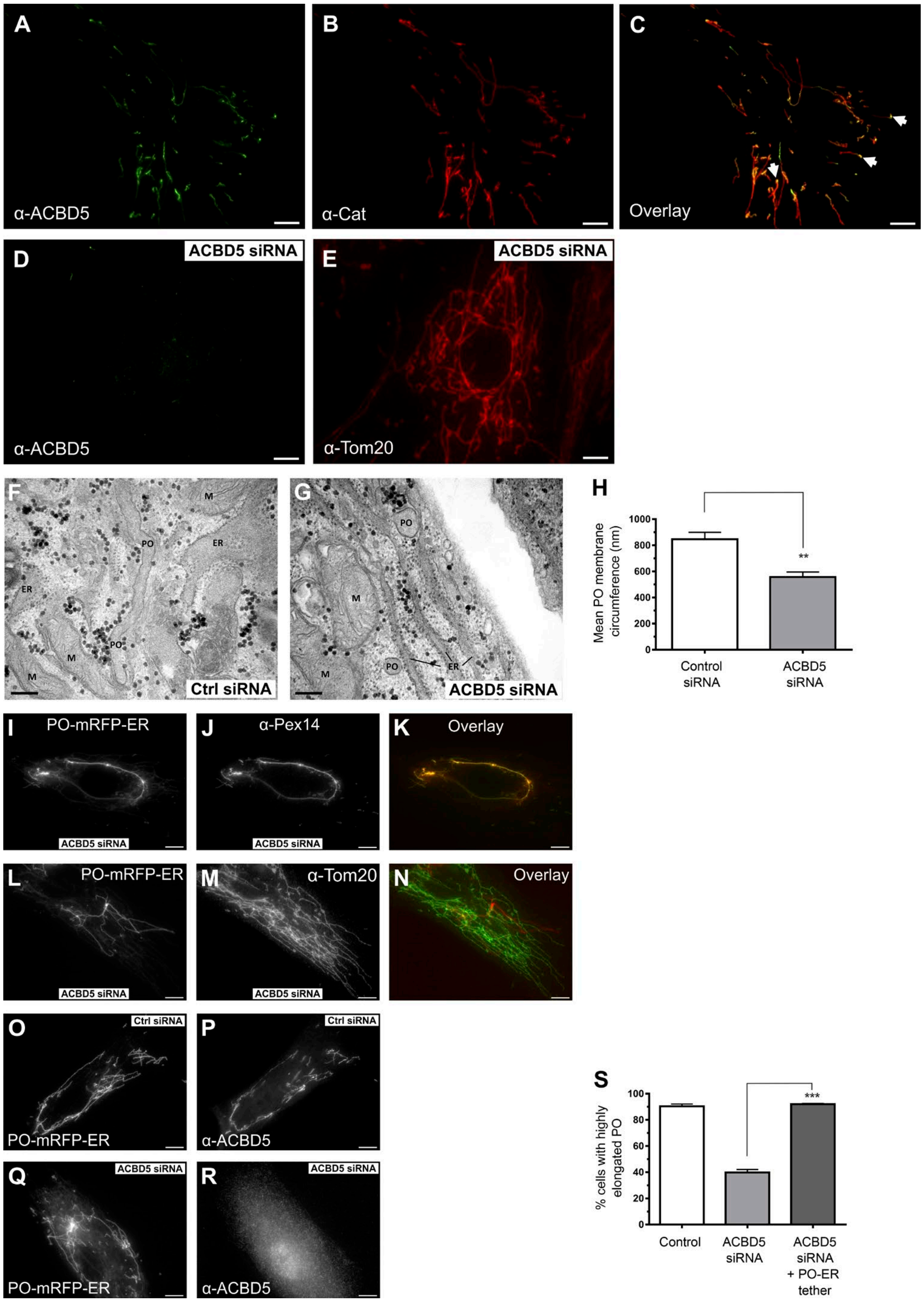

Figure S3. **PO membrane extension after ACBD5 knockdown in Mff-deficient fibroblasts can be rescued by expression of an artificial PO–ER tether.** (A–C) Endogenous ACBD5 associates with PO. Fixed Mff-deficient human skin fibroblasts were labeled with anti-ACBD5 and anti-catalase (peroxisomal marker) antibodies. Note that ACBD5 is concentrated at globular POs that give rise to tubular membranes (arrows). (D and E) Silencing of ACBD5 does not impact mitochondrial morphology. After siRNA mediated silencing of ACBD5, fixed Mff-deficient human skin fibroblasts were labeled with anti-ACBD5 and anti-TOM20 antibodies (mitochondrial marker). Note that ACBD5 silencing is efficient but mitochondrial morphology is not altered. (F–H) Silencing of ACBD5 reduces PO profile size in Mff-deficient fibroblasts. siRNA-treated cells were fixed and processed for EM and the mean PO membrane circumference of unbiasedly sampled PO profiles quantified by intersection counting (see Materials and methods). Electron micrographs of PO profiles in Mff-deficient cells treated with control (F) or ACBD5 siRNA (G). Bars, 200 nm. M, mitochondrion. (H) Assessment of the mean PO membrane circumference. Data tested for normal distribution by Lilliefors test then analyzed by two-tailed *t* test; **, $P \leq 0.01$. Error bars are SEM from six experiments per condition. (I–S) Expression of an artificial PO–ER tether restores membrane expansion in ACBD5-silenced Mff-deficient fibroblasts. An artificial PO–ER tether (PO-mRFP-ER) was generated based on a previously published MITO-ER tether (Csordás et al., 2006; see Materials and methods). Expression of PO-mRFP-ER in Mff-deficient, ACBD5 siRNA-treated fibroblasts restores membrane elongation of PO and results in hyperextension of PO membranes (I–L and Q). Note that some mistargeting to mitochondria is also observed (L–N). PO-mRFP-ER colocalizes with ACBD5 in controls (O and P) and promotes PO elongation in the absence of ACBD5 (Q and R; R, high intensity to highlight loss of ACBD5). (S) Quantification of PO morphology in controls, ACBD5-silenced cells, and ACBD5-silenced cells expressing the PO–ER tether (n = 500, three independent experiments). Data are presented as mean ± SEM. αPEX14, PO marker; α-Tom20, mitochondrial marker. Bars, 10 µm.

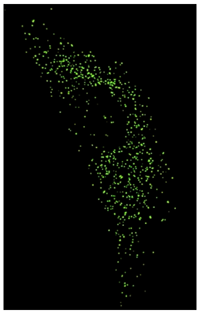

Video 1. **Loss of ACBD5 increases PO movement.** Human fibroblasts were transfected with mock (SV1) or ACBD5 siRNA (SV2) and GFP-PTS1 and analyzed by live-cell imaging using an IX81 microscope (Olympus) equipped with a CSUX1 spinning disk head (Yokogawa). 250 stacks of nine planes (0.5 µm thickness, 100 ms exposure) were taken in a continuous stream. 125 frames, 10× speed.

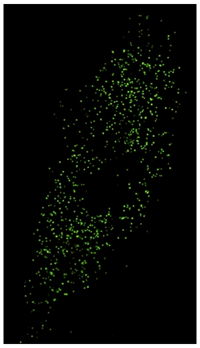

Video 2. **Loss of ACBD5 increases PO movement.** Human fibroblasts were transfected with mock (SV1) or ACBD5 siRNA (SV2) and GFP-PTS1 and analyzed by live-cell imaging using an IX81 microscope (Olympus) equipped with a CSUX1 spinning disk head (Yokogawa). 250 stacks of nine planes (0.5 µm thickness, 100 ms exposure) were taken in a continuous stream. 125 frames, 10× speed.

Table S1. **Plasmids used in this study**

Plasmid	Source
Myc-VAPB	C. Miller, King's College London, London, England, UK
pETM41	M. Wilmanns, European Molecular Biology Laboratory Hamburg, Hamburg, Germany
EGFP-Sec61β	W.A. Prinz, National Institutes of Health, Bethesda, MD
Myc-rACBD5.1	Islinger et al., 2007
EGFP-PTS1	Koch et al., 2005
FLAG-MFF	K. Mihara, Kyushu University, Fukuoka, Japan
mAKAP1 (34–63)-mRFP-yUBC6 (MITO–ER linker)	G. Hajnoczky, Thomas Jefferson University, Philadelphia, PA

Table S2. **Plasmids generated in this study**

Name	Template	Primers	Enzymes	Vector
EGFP-ACBD5.1	Myc-rACBD5.1	GFP-ACBD5.1-XhoI-Fw GFP-ACBD5.1-EcoRI-Rv	XhoI–EcoRI	pEGFP-C1
Myc-ACBD5	HepG2 cDNA	Myc-ACBD5 For Myc-ACBD5 Rev	EcoRV–XhoI	pCMV-3b
FLAG-ACBD5	Myc-ACBD5	—	EcoRV–XhoI	pCMV-2b
ACBD5_NcoIdel	Myc-ACBD5	ACBD5_NcoIdel_F ACBD5_NcoIdel_R		
MBP-ACBD5	ACBD5_NcoIdel	pETM41_ACBD5_For pETM41_ACBD5_Rev	NcoI–KpnI	pETM41
MBP-ACBD5ΔTMD	MBP-ACBD5	ACBD5_HS_S495X_F ACBD5_HS_S495X_R		
MBP-ACBD5 AcB	MBP-ACBD5ΔTMD	ACBD5_K78A_For ACBD5_K78A_Rev ACBD5_Y74F_For ACBD5_Y74F_Rev		
MBP-ACBD5 FFAT	MBP-ACBD5ΔTMD	ACBD5_C302K_For ACBD5_C302K_Rev ACBD5_S304E_For ACBD5_S304E_Rev ACBD5_Y301K_F ACBD5_Y301K_R		
GST-VAPB	Myc-VAPB	GST_VAPB_F GST_VAPB_R	BamHI–NotI	pGEX-6p2
GST-VAPBΔTMD	GST-VAPB	VAPB_220 For VAPB_220 Rev		
FLAG-ACBD5-AcB	FLAG-ACBD5	ACBD5_K78A_For ACBD5_K78A_Rev ACBD5_Y74F_For ACBD5_Y74F_Rev		
FLAG-ACBD5 FFAT	FLAG-ACBD5	ACBD5_C302K_For ACBD5_C302K_Rev ACBD5_S304E_For ACBD5_S304E_Rev ACBD5_Y301K_F ACBD5_Y301K_R		
EGFP-ACBD5-MITO	EGFP-ACBD5	ACBD5_M2SDM_F ACBD5_M2SDM_R		
Pex3 (1–44)-RFP-yUBC6 (PO–ER tether)	mAKAP1 (34–63)-mRFP-yUBC6	—	NheI–AgeI	First 132 bp of human Pex3 replacing mAKAP1 (34–63) in the MITO–ER tether

Table S3. **Primers used in this study**

Name	Sequence (5′ to 3′)
GFP-ACBD5.1-XhoI-Fw	CCGCTCGAGAAATGGCGGACACACGATCAG
GFP-ACBD5.1-EcoRI-Rv	CCGGAATTCCGTCAATTTAGTTTTCTTCTCCTTC
Myc-ACBD5 For	AAAGGATCCATGCTCTTCCTCTCGTTTCATG
Myc-ACBD5 Rev	GTTCTCGAGTTATCAGTTCAGTTTTCTTCTCCTTCTTTG
ACBD5_NcoIdel_F	GTCATTCCAGTCAACCTATGGAAAATTCTGG
ACBD5_NcoIdel_R	CCAGAATTTTCCATAGGTTGACTGGAATGAC
pETM41_ACBD5_For	TCACCATGGACATGCTCTTCCTCTCGTTTCATG
pETM41_ACBD5_Rev	CCTGGTACCTCAGTTCAGTTTTCTTCTCCTTCTTTG
ACBD5_HS_S495X_F	TCTTGGTGGCCCTTCGAGATGTGACCTGGTGTGCTAACGTTTGC
ACBD5_HS_S495X_R	GCAAACGTTAGCACACCAGGTCACATCTCGAAGGGCCACCAAGA
ACBD5_K78A_For	GCTTAAATTTTATAGCTTCTATGCGCAGGCAACTGAAGGACCCTG
ACBD5_K78A_Rev	CAGGGTCCTTCAGTTGCCTGCGCATAGAAGCTATAAAATTTAAGC
ACBD5_Y74F_For	GATGCTTAAATTTTTTAGCTTCTATGCGCAGGC
ACBD5_Y74F_Rev	GCCTGCGCATAGAAGCTAAAAAATTTAAGCATC
ACBD5_C302K_For	TCAGACAGTGAAGTTTACAAGGATTCTATGGAACAATTTG
ACBD5_C302K_Rev	CAAATTGTTCCATAGAATCCTTGTAAACTTCACTGTCTGA
ACBD5_S304E_For	AGTGAAGTTTACAAGGATCGTATGGAACAATTTGGACAAG
ACBD5_S304E_Rev	CTTGTCCAAATTGTTCCATACGATCCTTGTAAACTTCACT
ACBD5_Y301K_F	GATTCAGACAGTGAAGTTAAGAAGGATCGTATGGAAC
ACBD5_Y301K_R	GTTCCATACGATCCTTCTTAACTTCACTGTCTGAATC
VAPB_220 For	TGGGAAGGAAGAAGGCTAGAGCACCCGGCTCTTGG
VAPB_220 Rev	CCAAGAGCCGGGTGCTCTAGCCTTCTTCCTTCCCA
GST_VAPB_F	TCAGGATCCATGGCGAAGGTGGAGCAGGT
GST_VAPB_R	TTGGGAAGATTGCCTTGTAGGCGGCCGCATT
ACBD5_M2SDM_F	GTATTTATACTATCAAAGAGCGAGAGCAAAACTGAACTGACTCGAG
ACBD5_M2SDM_R	CTCGAGTCAGTTCAGTTTTGCTCTCGCTCTTTGATAGTATAAATAC

Table S4. **siRNAs used in this study**

Name	Sequence (5′ to 3′)	Source
ACBD5 siRNA	GCAUUCACCAAGAUAUAAATT	Ambion (s40666)
VAPB siRNA	GCUCUUGGCUCUGGUGGUUUU	Eurofins
Pex11β siRNA	AUUAGGGUGAGAAUAGACAGGAUGG	Eurofins
Control siRNA	si-GENOME nontargeting siRNA pool #2	GE Healthcare (D-001206-14-05)

Table S5. **Primary and secondary antibodies used in this study**

Antibodies	Type	Dilution		Source
		IMF	WB	
ACBD5 (HPA012145)	pc rb	1:150	1:250	Sigma-Aldrich
ACOX1	pc rb		1:10,000	T. Hashimoto, Shinshu University School of Medicine, Nagano, Japan
ATP synthase	mc ms		1:1,000	Abcam
Catalase	pc ms	1:150		Abcam
FLAG	mc ms	1:500		Sigma-Aldrich
FLAG	pc rb	1:750	1:1,000	Sigma-Aldrich
GAPDH	mc rb		1:5,000	ProSci
GFP (A11122)	pc rb		1:1,000	Thermo Fisher Scientific
Myc (Ab9106)	mc rb	1:200	1:1,000	Abcam
Myc (9E10)	mc ms	1:200	1:1,000	Santa Cruz Biotechnology, Inc.
PEX14	pc rb	1:1,400	1:4,000	D. Crane, Griffith University, Brisbane, Australia
PEX11β (ab 182100)	pc rb		1:1,000	Abcam
PDI	mc ms	1:100		Thermo Fisher Scientific
Thioredoxin (ab 179843)	mc rb		1:250	OXSD cocktail; Abcam
TOM20 (612278)	mc ms	1:200		BD
VAPB	pc rb		1:500	Sigma-Aldrich
Alexa Fluor 488 IgG	dk anti-rb	1:500		Molecular Probes
Alexa Fluor 488 IgG	dk anti-ms	1:400		Molecular Probes
Alexa Fluor 594 IgG	dk anti-rb	1:1,000		Molecular Probes
Alexa Fluor 594 IgG	dk anti-ms	1:1,000		Molecular Probes
TRITC IgG	dk anti-ms	1:100		Dianova
TRITC IgG	dk anti-rb	1:400		Dianova
HRP IgG	gt anti-ms		1:5,000	Bio-Rad Laboratories
HRP IgG	gt anti-rb		1:5,000	Bio-Rad Laboratories

dk, donkey; gt, goat; mc, monoclonal; ms, mouse; pc, polyclonal; rb, rabbit.

References

Csordás, G., C. Renken, P. Várnai, L. Walter, D. Weaver, K.F. Buttle, T. Balla, C.A. Mannella, and G. Hajnóczky. 2006. Structural and functional features and significance of the physical linkage between ER and mitochondria. *J. Cell Biol.* 174:915–921. http://dx.doi.org/10.1083/jcb.200604016

Islinger, M., G.H. Lüers, K.W. Li, M. Loos, and A. Völkl. 2007. Rat liver peroxisomes after fibrate treatment. A survey using quantitative mass spectrometry. *J. Biol. Chem.* 282:23055–23069. http://dx.doi.org/10.1074/jbc.M610910200

Koch, A., Y. Yoon, N.A. Bonekamp, M.A. McNiven, and M. Schrader. 2005. A role for Fis1 in both mitochondrial and peroxisomal fission in mammalian cells. *Mol. Biol. Cell.* 16:5077–5086. http://dx.doi.org/10.1091/mbc.E05-02-0159
